# Supplementary material for: LRRTM2 controls presynapse nano-organization and AMPA receptor sub-positioning through Neurexin-binding interface
Source: Nat Commun. 2024 Oct 11;15:8807. doi: 10.1038/s41467-024-53090-y (PMC11470055; doi:10.1038/s41467-024-53090-y)
Supplement: Supplementary file 1 — Supplementary Information [file 41467_2024_53090_MOESM1_ESM.pdf]

Supplementary Figures

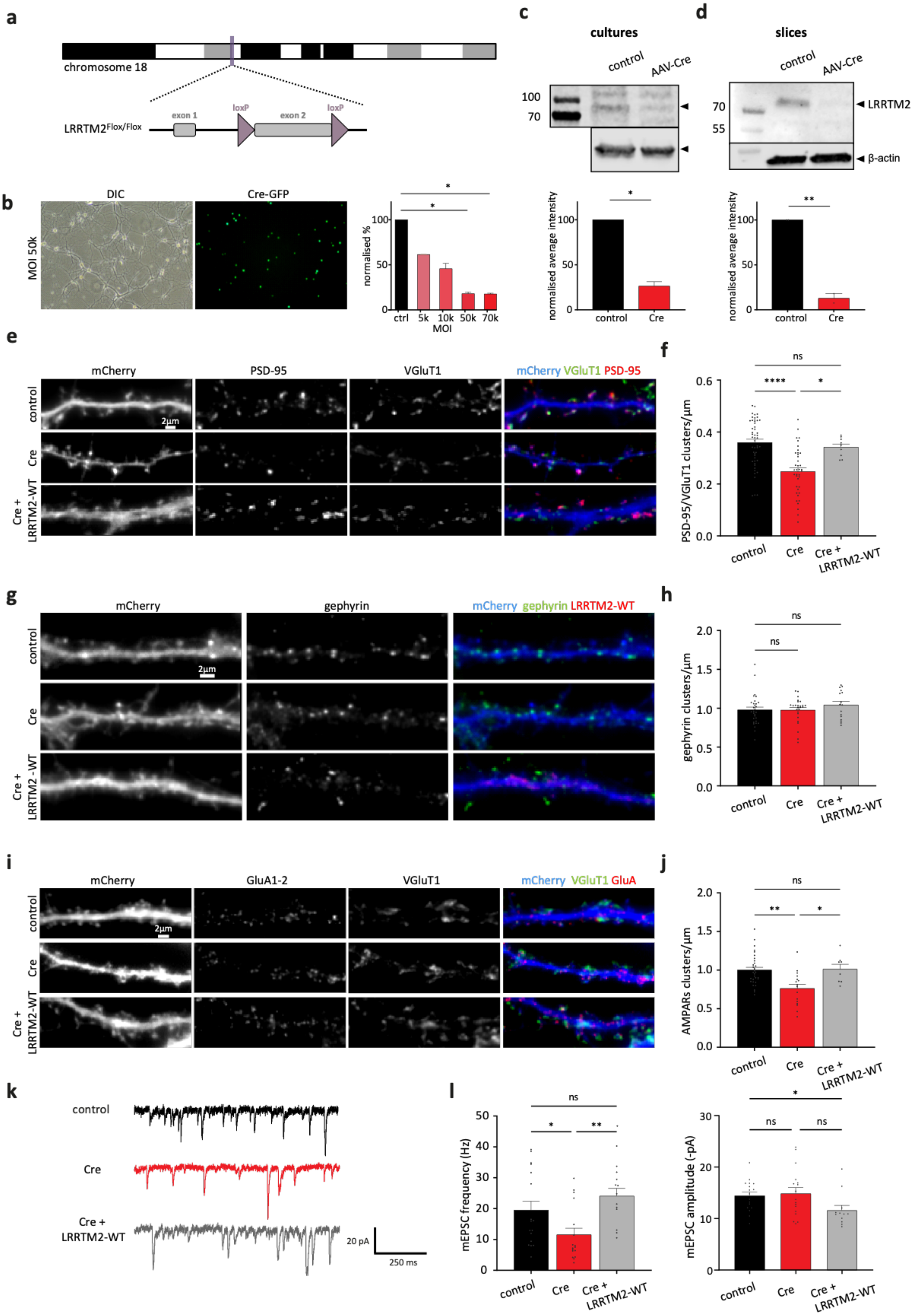

Supplementary Figure 1. Conditional knock-out of LRRTM2 impairs excitatory synapse development and function.

(a) Schematics of LRRTM2 gene locus in mouse chromosome 18. Purple inset shows the 2 exons of LRRTM2 gene and the insertion of loxP sites upstream and downstream of exon 2. (b) Representative images of cultured hippocampal neurons from LRRTM2$^{Flox/Flox}$ mice infected with nuclear Cre-GFP-encoding virus and RT-qPCR quantification of LRRTM2 RNA expression in control (control) or in neurons infected with increasing dose of Cre-GFP virus (MOI: 5k, 10k, 20k, 50k and 70k). Data acquired from 2 experiments for each condition. MOI: multiplicity of infection, * p<0.05. (c) LRRTM2 and β-actin immunoblots of proteins extracted from hippocampal LRRTM2$^{Flox/Flox}$ cultures. Data presented as mean +/- sem, obtained from 3 independent experiments, * p<0.05, compared by unpaired t-test. (d) LRRTM2 and β-actin immunoblots of proteins extracted from organotypic hippocampal slices not infected (control) or infected with an AAV-Cre virus (AAV-Cre). Data presented as mean +/- sem, obtained from 2 experiments ** p<0.01 compared by unpaired t-test. (e) DIV15 hippocampal neurons expressing mCherry (control), Cre-mCherry (Cre) or Cre-mCherry and biotinylated AP-LRRTM2 (Cre+LRRTM2-WT) immunostained for endogenous PSD-95 and VGlut1 as post- and pre-synaptic markers, respectively. Right, Cre-mCherry (blue) overlaid with PSD-95 (red) and VGluT1 (green). (f) Quantification of synaptic density measured as the apposition of PSD-95 and VGluT1 puncta. Data acquired from 3 (control: n=49, Cre: n=38 cells) and 2 independent experiments (Cre + LRRTM2-WT: n=9 cells) * p<0.05, **** p<0.0001. (g) DIV15 hippocampal neurons expressing mCherry (control), Cre-mCherry (Cre) or Cre-mCherry and biotinylated AP-LRRTM2 (Cre + LRRTM2-WT) immunostained for endogenous gephyrin as marker of inhibitory synapses. Right, Cre-mCherry (blue) overlaid with gephyrin (green) and for the Cre + LRRTM2-WT condition, with AP-LRRTM2 (red). (h) Quantification of inhibitory synaptic density measured as the number of gephyrin clusters/µm. Data acquired from 2 independent experiments (control: n=30, Cre: n=26, Cre + LRRTM2-WT=16 cells). (i) DIV15 hippocampal neurons expressing mCherry (control), Cre-mCherry (Cre) or Cre-mCherry and biotinylated AP-LRRTM2 (Cre + LRRTM2-WT) immunostained for endogenous GluA1/2 subunits of AMPARs and VGluT1. Right, Cre-Cherry (blue) overlaid with GluA1/2 (red) and VGluT1 (green). (j) Quantification of normalized AMPARs density showing significant decrease of AMPAR cluster density in the absence of LRRTM2. Data acquired from 3 independent experiments (control: n=31, Cre: n=16, Cre + LRRTM2-WT=8 cells), * p<0.05, ** p<0.01. (k) Representative mEPSC traces recorded from DIV15 hippocampal neurons expressing mCherry (control), Cre-mCherry (Cre) or Cre-mCherry and biotinylated AP-LRRTM2 (Cre + LRRTM2-WT). (l) mean mEPSC frequency for control, Cre, and Cre + LRRTM2-WT showing a decreased frequency, but no change in mEPSC amplitude in the cKO compared to control condition. Data acquired from 3 (control: n=17, Cre: n=17 cells) and 2 independent experiments (Cre + LRRTM2-WT n=13 cells), * p<0.05, **p<0.01. Data are presented as mean values +/- SEM. (b,f,j,l) Data were compared by one-way analysis of variance test, followed by post-hoc Dunn's test.

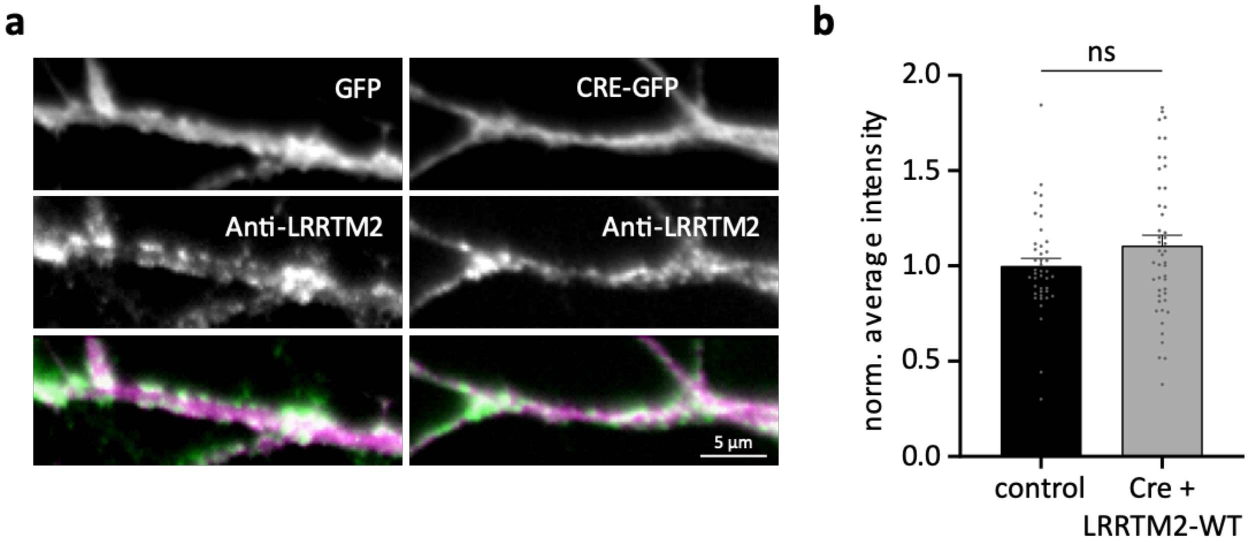

Supplementary Figure 2. LRRTM2 expression on cKO background is similar to endogenous levels.

(a) DIV15 hippocampal neurons expressing GFP (control) or Cre-GFP and biotinylated AP-LRRTM2 (Cre + LR-RTM2-WT) immunostained for LRRTM2 after glyoxal fixation and (b) normalized average intensity of LRRT-M2 expression in each conditions, showing no significant change in protein expression. Data acquired from 3 independent experiments (control, n=42 cells, Cre + LRRTM2-WT n=47 cells). Data are presented as mean values +/- SEM. Data were compared by non-parametric Mann-Whitney test.

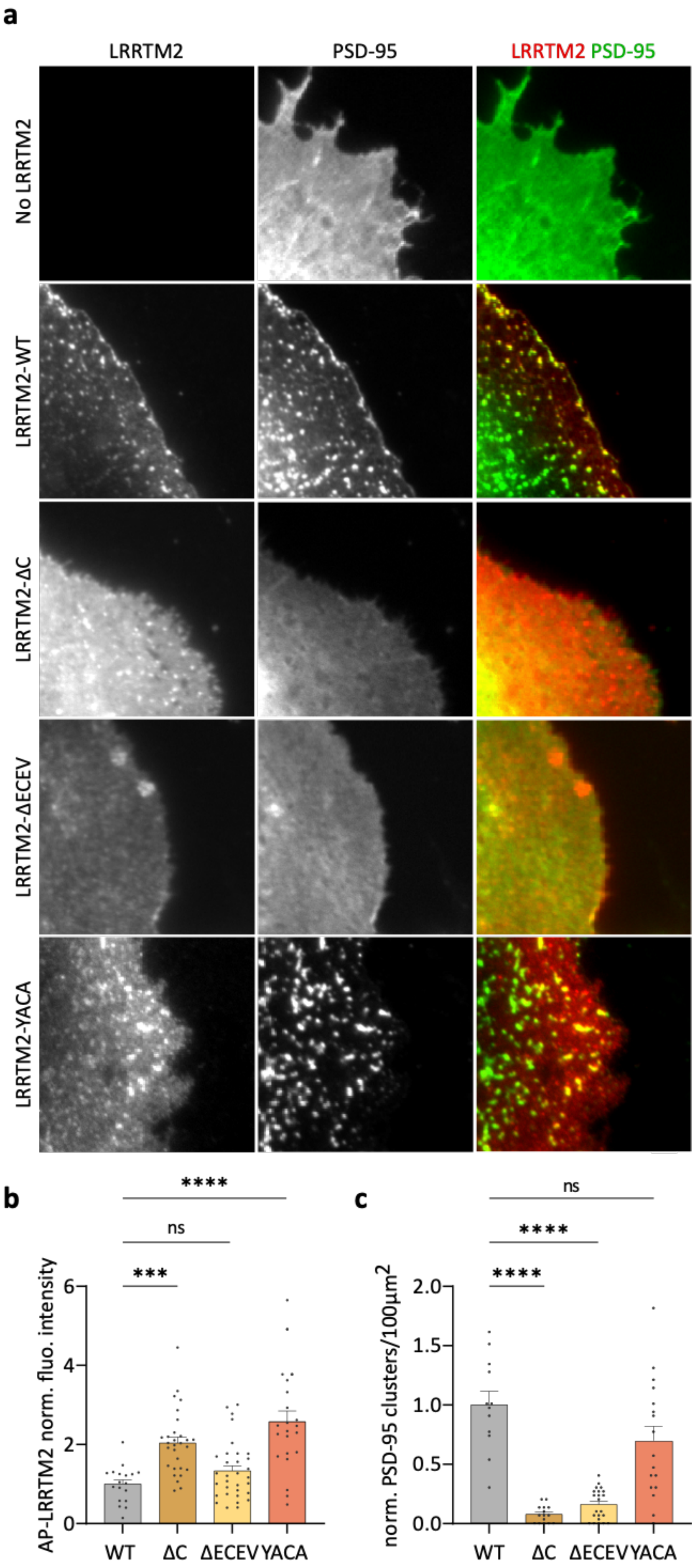
a
LRRTM2
PSD-95
LRRTM2 PSD-95
No LRRTM2
LRRTM2-WT
LRRTM2-ΔC
LRRTM2-ΔECEV
LRRTM2-YACA
b
AP-LRRTM2 norm. fluo. intensity
****
ns
***
WT
ΔC
ΔECEV
YACA
c
norm. PSD-95 clusters/100µm2
ns
****
****
WT
ΔC
ΔECEV
YACA

Supplementary Figure 3. LRRTM2 and PSD-95 co-cluster through the ECEV motif.

(a) COS-7 cells expressing intracellular mutants of AP-LRRTM2, BirA^{ER}, and PSD-95-GFP (middle) surface labeled with ATTO565-conjugated monomeric streptavidin, ATTO565-mSA (left). Notice the homogeneous distribution of PSD-95 in the absence of LRRTM2 (top). On the right, AP-LRRTM2 mutants (red) are overlaid with PSD-95-GFP (green). (b) Normalized fluoresce intensity of AP-LRRTM2 mutants showing an increase in surface expression for the ΔC and the YACA. (c) Quantification of the PSD-95 density per cell for the different conditions showing that LRRTM2 and PSD-95 co-cluster through the intracellular ECEV motif. Data obtained from 2 experiments (WT: n=12; ΔC n=19; ΔECEV: n=23; YACA: n=17 cells), *** $p<0.001$, **** $p<0.0001$. Data are presented as mean values +/- SEM. Data were compared by one-way analysis of variance test, followed by post hoc Dunn's test.

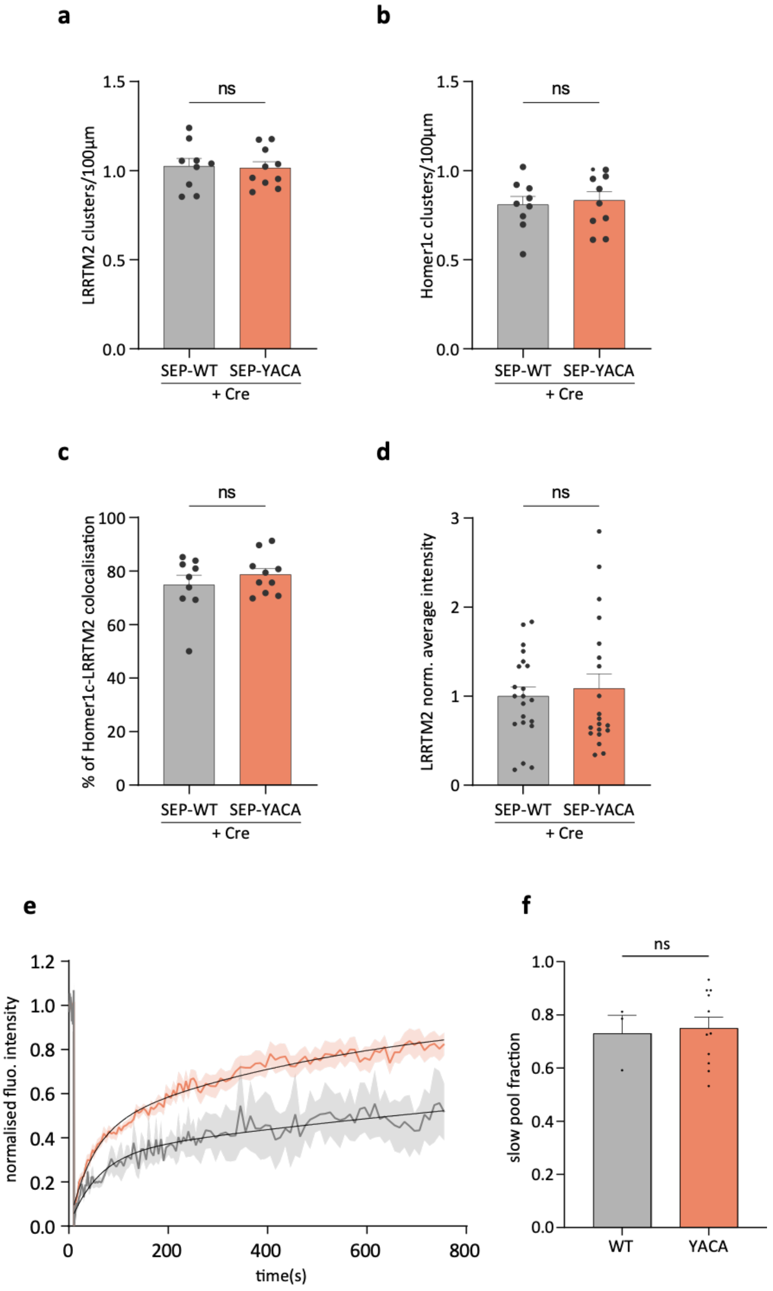

Supplementary Figure 4. SEP-LRRTM2-YACA and SEP-LRRTM2-WT are equally localized to synapses.

(a) Quantification of SEP-LRRTM2-WT and SEP-LRRTM2-YACA cluster density. (b) Quantification of Homer1c-BFP cluster density for SEP-LRRTM2-WT and SEP-LRRTM2-YACA. (c) Percentage of Homer1c-BFP and SEP-LRRTM2-WT or SEP-LRRTM2-YACA colocalization. Data obtained from 3 independent experiments (SEP-LRRTM2-WT n=9; SEP-LRRTM2-YACA n=10 cells). (d) Normalized average intensity of SEP-WT and SEP-YACA. Data obtained from 3 independent experiments (SEP-LRRTM2-WT n=21; SEP-LRRTM2-YACA n=20 cells). (e) FRAP recovery curves outside synapses and (f) corresponding slow pool fractions extracted from FRAP curve fitting. Data acquired from 3 independent experiments (SEP-LRRTM2-WT n=3 regions, SEP-LRRTM2-YACA n=11 regions). Data are presented as mean values +/- SEM. Data were compared by non-parametric Mann-Whitney test.

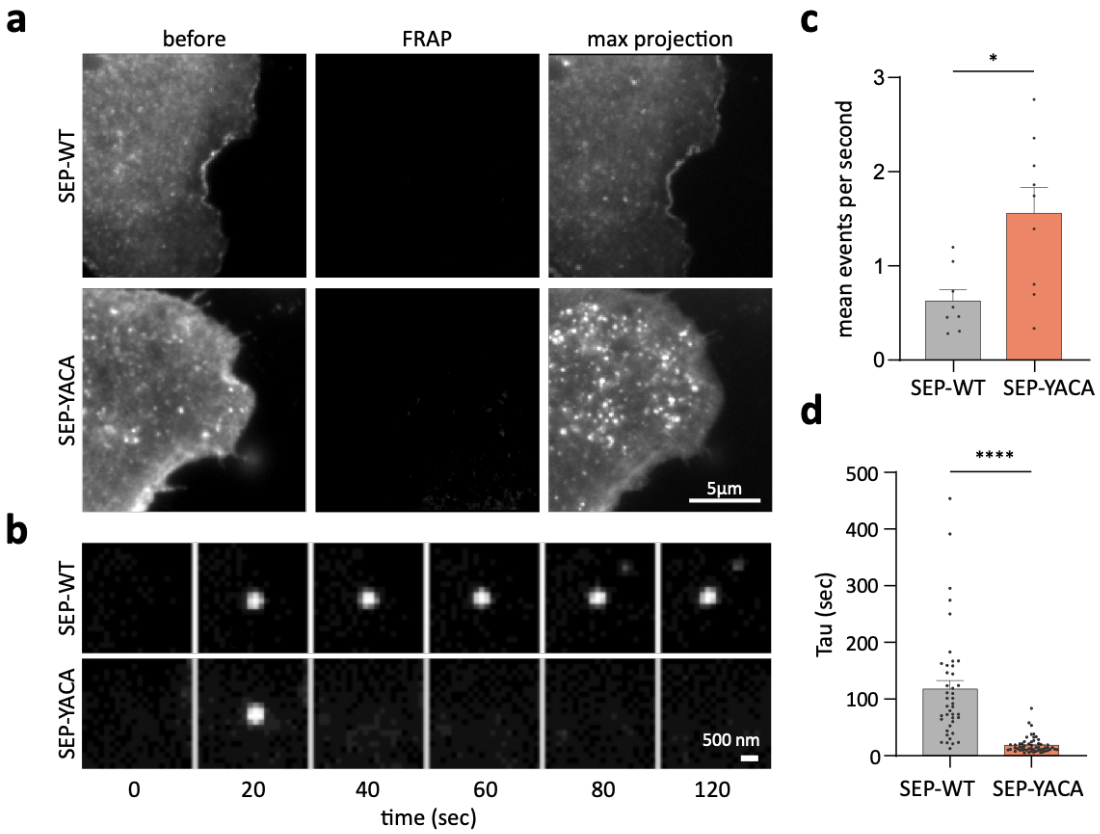

Supplementary Figure 5. The C-terminal YxxC motif regulates LRRTM2 exocytosis in COS-7 cells.

(a) Representative images of COS-7 cells expressing SEP-LRRTM2-WT or SEP-LRRTM2-YACA (left). After photobleaching of all surface proteins (middle), individual exocytic events were monitored with time-lapse imaging. Maximum projection of the frames acquired is depicted in the right panel, showing drastic increase in exocytic events in the SEP-LRRTM2-YACA condition. (b) Examples of individual exocytic events of SEP-LRRTM2-WT and SEP-LRRTM2-YACA. (c) Quantification of the mean number of exocytic events per second per field of view. (d) Rate of fluorescence decay of individual exocytic events. Data acquired from 2 experiments. SEP-LRRTM2-WT: n=8 cells, SEP-LRRTM2-YACA: n=9 cells, * $p<0.05$, **** $p<0.0001$. Data are presented as mean values +/- SEM. Data were compared by non-parametric Mann-Whitney test.

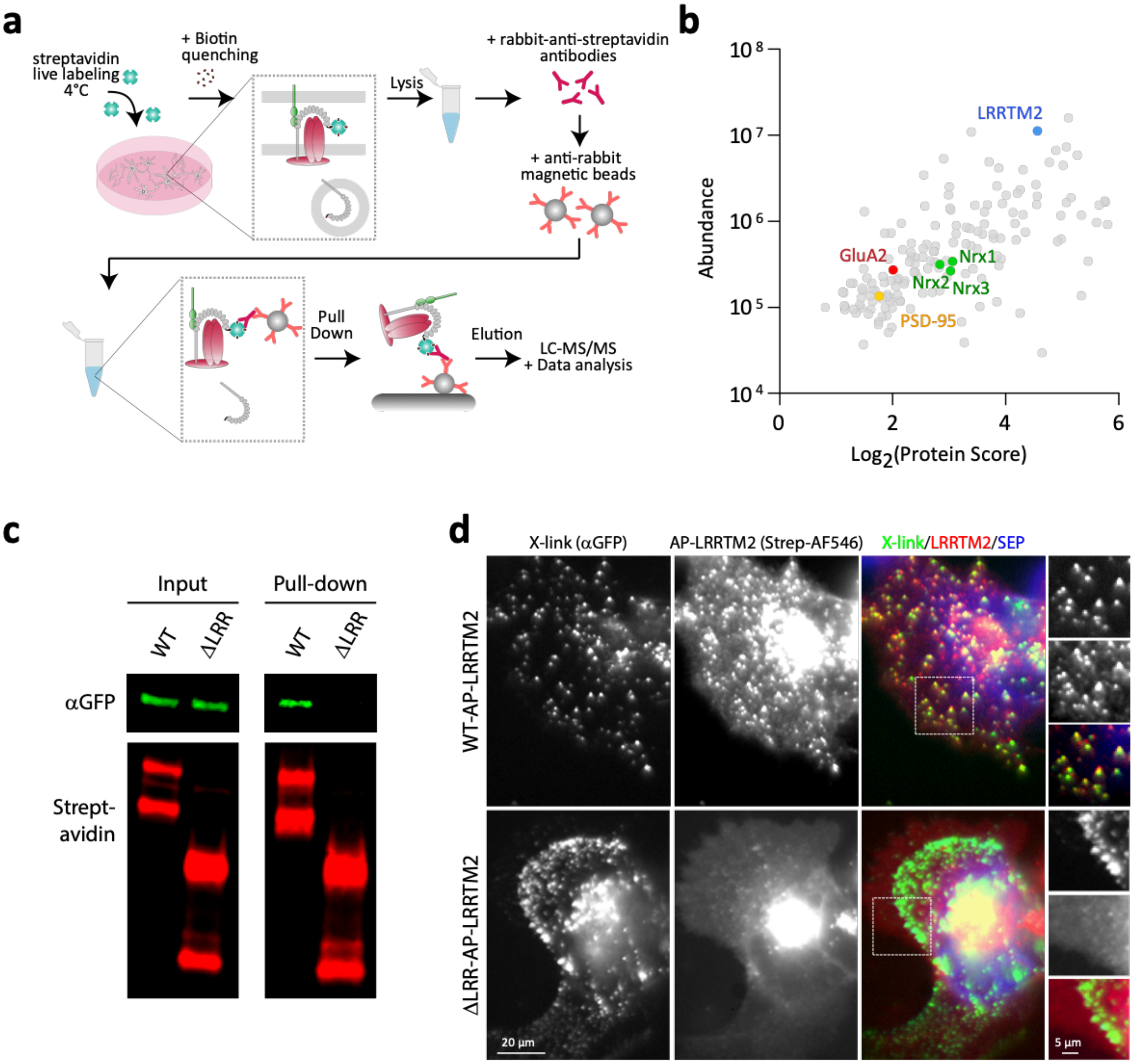

Supplementary Figure 6. AMPARs are present in LRRTM2 surface proteome.

(a) Schematics showing the strategy for selective targeting of LRRTM2 surface interactome. Cells were incubated with streptavidin 40 min at 4°C to prevent internalization, and quenched with biotin (5 min) before lysis. Anti-streptavidin antibodies were used to target surface-streptavidin-labeled LRRTM2 followed by incubation with anti-rabbit-coated magnetic bead incubation OVN at 4°C. A magnetic holder was used to immobilized pulled-down beads for washes, before sample elution and LC-MS/MS analysis (b) Mass spectrometry interaction screen showing the presence of AMPAR subunit GluA2 in the surface proteome of LRRTM2. The data are plotted as a measure of protein abundance versus protein score calculated as the sum of the negative log of the posterior error probability of connected peptide-spectrum matches. Each dot in the scatter plot represents a protein identified by mass spectrometry. The blue dot is LRRTM2 (bait protein), the green dots are Neurexin family members 1-3, known to interact with LRRTM2, the yellow dot represents PSD-95, also known interactor of LRRTM2, and the red dot represents GluA2. Gray dots represent other proteins that were also identified (see Table S1). (c) Representative Western blot showing input and co-precipitation of SEP-GluA2 by streptavidin pull-down in COS-7 cells expressing SEP-GluA2, $BirA^{ER}$, and WT-AP-LRRTM2 or ΔLRR-AP-LRRTM2. SEP-GluA2 co-precipitates in the presence of WT-LRRTM2 but not ΔLRR-LRRTM2 (n=2 independent experiments). (d) Representative images of live co-recruitment assays in COS-7 cells expressing SEP-GluA2, $BirA^{ER}$, and WT-AP-LRRTM2 or ΔLRR-AP-LRRTM2 (n=2 independent experiments, 30 cells per condition). SEP-GluA2 was cross-linked with secondary and primary antibodies against GFP, and LRRTM2 co-recruitment to these clusters was assessed by live imaging. WT-LRRTM2, but not ΔLRR-LRRTM2 co-segregated with GluA2.

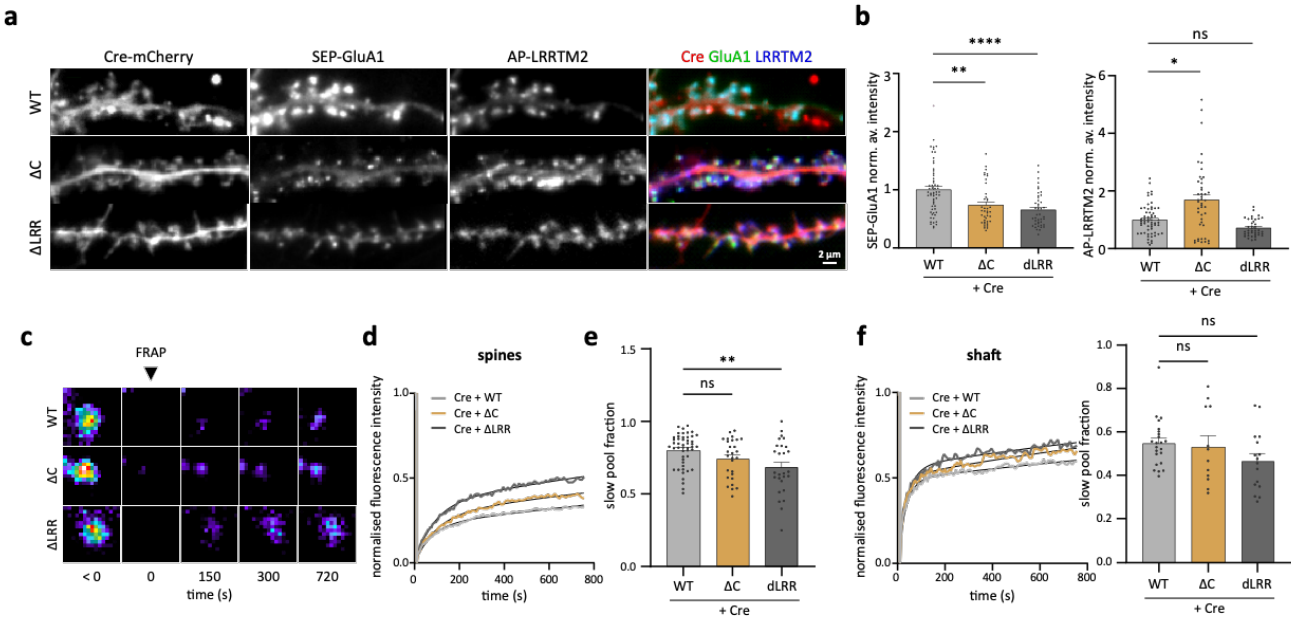

Supplementary Figure 7. AP-LRRTM2 controls AMPARs stabilization via its extracellular region.

(a) DIV15 hippocampal neurons expressing Cre-mCherry, BirA[ER], SEP-GluA1 and LRRTM2-WT (WT), LRRTM2-ΔC (ΔC) or LRRTM2-ΔLRR (ΔLRR). On the right, mCherry (red) is overlaid with SEP-GluA1 (green) and the different LRRTM2 constructs (blue). (b) Normalized expression levels of SEP-GluA1 in FRAP regions, and normalized surface intensity of AP-LRRTM2 in each condition. (c) FRAP experiments on spine-accumulated SEP-GluA1 expressing Cre-mCherry and AP-LRRTM2 mutants showing a faster recovery in the ΔLRR condition. (d) Normalized fluorescence recovery curves in spines showing an intermediate recovery for the ΔC, and (e) corresponding slow pool fraction extracted from FRAP curve fitting, showing a reduction of the slow pool fraction only in the ΔLRR condition. (f) Normalized fluorescence recovery curves in shaft regions showing no difference in the recovery rates or in the slow pool fractions. Data obtained from 3 independent experiments. (spines WT: 51; ΔC: 28; ΔLRR: 27 regions; shaft WT: 23; ΔC: 12; ΔLRR: 16 regions), * $p<0.05$, ** $p<0.01$, **** $p<0.0001$. Data are presented as mean values +/- SEM. Data were compared by one-way analysis of variance test, followed by post hoc Dunn's test.

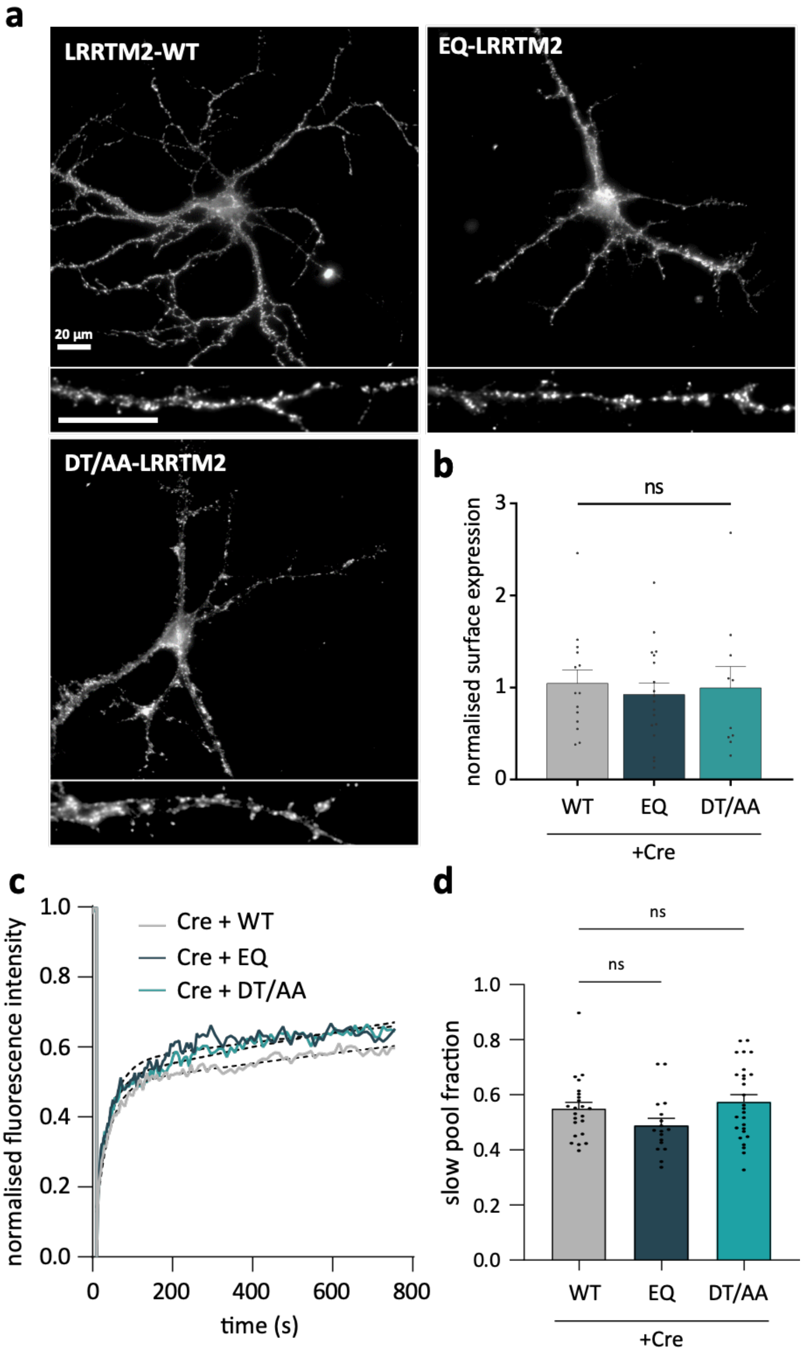

Supplementary Figure 8. LRRTM2 extracellular mutants display similar expression levels.

(a) Representative images of DIV15 hippocampal neurons expressing Cre-mCherry, BirA^{ER} and biotinylated AP-LRRTM2 (WT, EQ or DT/AA). Insets depict labeling of LRRTM2 mutants at the surface of individual dendrites. (b) Normalized fluorescence intensity showing no change in expression of the mutants at the plasma membrane of neurons. Data obtained from 2 independent experiments (WT: 14, EQ: n= 18, DT/AA: n= 10 cells). (c) Normalized fluorescence intensity of fluorescence recovery for SEP-GluA1 in regions outside synapses and (d) corresponding slow pool fractions of SEP-GluA1 extracted from FRAP curve fitting showing no differences between conditions. Data are presented as mean values +/- SEM. Data were compared by one-way analysis of variance test, followed by post hoc Dunn's test.

Gene Symbol	Description	Accession
**Tuba1a**	Tubulin alpha-1A	P68369
**Myh9**	Myosin-9	Q8VDD5
**Tuba4a**	Tubulin alpha-4A	P68368
**Myh10**	Myosin-10	Q61879
**Tubb5**	Tubulin beta-5	P99024
**Tubb2a**	Tubulin beta-2A	Q7TMM9
**Tubb2b**	Tubulin beta-2B	Q9CWF2
**Tubb4b**	Tubulin beta-4B	P68372
**Tubb3**	Tubulin beta-3	Q9ERD7
**Camk2a**	Calcium/calmodulin-dependent protein kinase type II subunit alpha	P11798
**Atp1a3**	Sodium/potassium-transporting ATPase subunit alpha-3	Q6PIC6
**Atp1a1**	Sodium/potassium-transporting ATPase subunit alpha-1	Q8VDN2
**Atp5f1a**	ATP synthase subunit alpha, mitochondrial	Q03265
**Lrrtm2**	Leucine-rich repeat transmembrane neuronal protein 2	Q8BGA3
**Eno1**	Alpha-enolase	P17182
**Actg1**	Actin, cytoplasmic 2	P63260
**Actb**	Actin, cytoplasmic 1	P60710
**Ywhaz**	14-3-3 protein zeta/delta	P63101
**Slc25a4**	ADP/ATP translocase 1	P48962
**Atp1a2**	Sodium/potassium-transporting ATPase subunit alpha-2	Q6PIE5
**Ywhaq**	14-3-3 protein theta	P68254
**Atp5f1b**	ATP synthase subunit beta, mitochondrial	P56480
**Ywhag**	14-3-3 protein gamma	P61982
**Gfap**	Glial fibrillary acidic protein	P03995
**Hsp90ab1**	Heat shock protein HSP 90-beta	P11499
**Prdx6**	Peroxiredoxin-6	O08709
**Nsf**	Vesicle-fusing ATPase	P46460
**Ywhah**	14-3-3 protein eta	P68510
**Slc25a5**	ADP/ATP translocase 2	P51881
**Gstm1**	Glutathione S-transferase Mu 1	P10649
**Basp1**	Brain acid soluble protein 1	Q91XV3
**Pkm**	Pyruvate kinase PKM	P52480
**Trim21**	E3 ubiquitin-protein ligase TRIM21	Q62191
**Hspa8**	Heat shock cognate 71 kDa protein	P63017
**Sptan1**	Spectrin alpha chain, non-erythrocytic 1	P16546
**Atp6v1b2**	V-type proton ATPase subunit B, brain isoform	P62814
**Myh14**	Myosin-14	Q6URW6
**Hsp90aa1**	Heat shock protein HSP 90-alpha	P07901
**Ywhab**	14-3-3 protein beta/alpha	Q9CQV8
**Glud1**	Glutamate dehydrogenase 1, mitochondrial	P26443
**Ckb**	Creatine kinase B-type	Q04447
**Ppp3ca**	Serine/threonine-protein phosphatase 2B catalytic subunit alpha isoform	P63328
**Tpi1**	Triosephosphate isomerase	P17751
**Gpi**	Glucose-6-phosphate isomerase	P06745
**Pdhx**	Pyruvate dehydrogenase protein X component, mitochondrial	Q8BKZ9
**Itm2c**	Integral membrane protein 2C	Q91VK4
**Sptbn1**	Spectrin beta chain, non-erythrocytic 1	Q62261
**Coro1c**	Coronin-1C	Q9WUM4
**Hspa2**	Heat shock-related 70 kDa protein 2	P17156

Gene Symbol	Description	Accession
**Pdia3**	Protein disulfide-isomerase A3	P27773
**Fscn1**	Fascin	Q61553
**Nrxn2**	Neurexin-2	E9Q7X7
**C1qb**	Complement C1q subcomponent subunit B	P14106
**Uba52**	Ubiquitin-60S ribosomal protein L40	P62984
**Camk2b**	Calcium/calmodulin-dependent protein kinase type II subunit beta	P28652
**C1qa**	Complement C1q subcomponent subunit A	P98086
**C1qc**	Complement C1q subcomponent subunit C	Q02105
**Atp1b1**	Sodium/potassium-transporting ATPase subunit beta-1	P14094
**Dbn1**	Drebrin	Q9QXS6
**Eef1g**	Elongation factor 1-gamma	Q9D8N0
**Rab3a**	Ras-related protein Rab-3A	P63011
**Dpysl3**	Dihydropyrimidinase-related protein 3	Q62188
**Gapdh**	Glyceraldehyde-3-phosphate dehydrogenase	P16858
**Aldoa**	Fructose-bisphosphate aldolase A	P05064
**Nrxn1**	Neurexin-1	Q9CS84
**Nrxn3**	Neurexin-3	Q6P9K9
**Actr3**	Actin-related protein 3	Q99JY9
**Phb**	Prohibitin	P67778
**Aldh2**	Aldehyde dehydrogenase, mitochondrial	P47738
**Aco2**	Aconitate hydratase, mitochondrial	Q99KI0
**Eef1a1**	Elongation factor 1-alpha 1	P10126
**Dnajb6**	DnaJ homolog subfamily B member 6	O54946
**Hspa1a**	Heat shock 70 kDa protein 1A	Q61696
**Eno2**	Gamma-enolase	P17183
**Ywhae**	14-3-3 protein epsilon	P62259
**Slc25a3**	Phosphate carrier protein, mitochondrial	Q8VEM8
**Rab3c**	Ras-related protein Rab-3C	P62823
**Gstm5**	Glutathione S-transferase Mu 5	P48774
**Uchl1**	Ubiquitin carboxyl-terminal hydrolase isozyme L1	Q9R0P9
**Prdx1**	Peroxiredoxin-1	P35700
**Ppm1h**	Protein phosphatase 1H	Q3UYC0
**Pygb**	Glycogen phosphorylase, brain form	Q8CI94
**Mdh2**	Malate dehydrogenase, mitochondrial	P08249
**Idh3a**	Isocitrate dehydrogenase [NAD] subunit alpha, mitochondrial	Q9D6R2
**Dsp**	Desmoplakin	E9Q557
**Vdac3**	Voltage-dependent anion-selective channel protein 3	Q60931
**Rap1gds1**	Rap1 GTPase-GDP dissociation stimulator 1	E9Q912
**Rac3**	Ras-related C3 botulinum toxin substrate 3	P60764
**Hspa5**	Endoplasmic reticulum chaperone BiP	P20029
**Atp2b1**	Plasma membrane calcium-transporting ATPase 1	G5E829
**Hk1**	Hexokinase-1	P17710
**Elavl1**	ELAV-like protein 1	P70372
**Aldoc**	Fructose-bisphosphate aldolase C	P05063
**Sdha**	Succinate dehydrogenase [ubiquinone] flavoprotein subunit, mitochondrial	Q8K2B3
**Vcp**	Transitional endoplasmic reticulum ATPase	Q01853
**Gria2**	Glutamate receptor 2	P23819
**Glul**	Glutamine synthetase	P15105
**Atp6v1h**	V-type proton ATPase subunit H	Q8BVE3

Gene Symbol	Description	Accession
**Myo5a**	Unconventional myosin-Va	Q99104
**Oxct1**	Succinyl-CoA:3-ketoacid coenzyme A transferase 1, mitochondrial	Q9D0K2
**Pdha1**	Pyruvate dehydrogenase E1 component subunit alpha, somatic form, mitochondrial	P35486
**C3**	Complement C3	P01027
**Rab10**	Ras-related protein Rab-10	P61027
**Pebp1**	Phosphatidylethanolamine-binding protein 1	P70296
**Psmb6**	Proteasome subunit beta type-6	Q60692
**Pcmt1**	Protein-L-isoaspartate(D-aspartate) O-methyltransferase	P23506
**Syngr1**	Synaptogyrin-1	O55100
**Psma5**	Proteasome subunit alpha type-5	Q9Z2U1
**Slc12a5**	Solute carrier family 12 member 5	Q91V14
**Dbt**	Lipoamide acyltransferase component of branched-chain alpha-keto acid dehydrogenase	P53395
**Mdh1**	Malate dehydrogenase, cytoplasmic	P14152
**C4b**	Complement C4-B	P01029
**Syngr3**	Synaptogyrin-3	Q8R191
**Stxbp1**	Syntaxin-binding protein 1	O08599
**Cct2**	T-complex protein 1 subunit beta	P80314
**Vdac1**	Voltage-dependent anion-selective channel protein 1	Q60932
**Atp2b2**	Plasma membrane calcium-transporting ATPase 2	Q9R0K7
**Chchd3**	MICOS complex subunit Mic19	Q9CRB9
**Mri1**	Methylthioribose-1-phosphate isomerase	Q9CQT1
**Prdx2**	Peroxiredoxin-2	Q61171
**Hadha**	Trifunctional enzyme subunit alpha, mitochondrial	Q8BMS1
**Hspa9**	Stress-70 protein, mitochondrial	P38647
**Crym**	Ketimine reductase mu-crystallin	O54983
**Crmp1**	Dihydropyrimidinase-related protein 1	P97427
**Pdhb**	Pyruvate dehydrogenase E1 component subunit beta, mitochondrial	Q9D051
**Tkt**	Transketolase	P40142
**Dlg4**	Disks large homolog 4	Q62108
**Myl12b**	Myosin regulatory light chain 12B	Q3THE2
**Got2**	Aspartate aminotransferase, mitochondrial	P05202
**Anxa1**	Annexin A1	P10107
**Rab7a**	Ras-related protein Rab-7a	P51150
**Stub1**	E3 ubiquitin-protein ligase CHIP	Q9WUD1
**Gnb1**	Guanine nucleotide-binding protein G(I)/G(S)/G(T) subunit beta-1	P62874
**Tmem109**	Transmembrane protein 109	Q3UBX0
**Hspd1**	60 kDa heat shock protein, mitochondrial	P63038
**Hsp90b1**	Endoplasmin	P08113
**Kif5b**	Kinesin-1 heavy chain	Q61768
**Septin8**	Septin-8	Q8CHH9
**Eef2**	Elongation factor 2	P58252
**Ddah1**	N(G),N(G)-dimethylarginine dimethylaminohydrolase 1	Q9CWS0
**Anxa7**	Annexin A7	Q07076
**Rab5a**	Ras-related protein Rab-5A	Q9CQD1
**Septin11**	Septin-11	Q8C1B7
**Ap2m1**	AP-2 complex subunit mu	P84091
**Sdhb**	Succinate dehydrogenase [ubiquinone] iron-sulfur subunit, mitochondrial	Q9CQA3
**Pacsin1**	Protein kinase C and casein kinase substrate in neurons protein 1	Q61644

Supplementary Table 1. List of proteins identified in Mass spectrometry interaction screen.

List of proteins identified in the proteomics dataset corresponding to Supplementary Figue 6b. Gene symbol, description, and accession numbers are provided. Note the presence of all known molecular interactors of LRRTM2 (Neurexins1-3 and Dlg4) along with GluA2 subunit of AMPARs.
