# Supplementary material for: LRRTM2 controls presynapse nano-organization and AMPA receptor sub-positioning through Neurexin-binding interface
Source: Nat Commun. 2024 Oct 11;15:8807. doi: 10.1038/s41467-024-53090-y (PMC11470055; doi:10.1038/s41467-024-53090-y)
Supplement: Supplementary file 3 — Reporting Summary [file 41467_2024_53090_MOESM3_ESM.pdf]

nature portfolio

Corresponding author(s): CHAMMA Ingrid

Last updated by author(s): Aug 28, 2024

Reporting Summary

Nature Portfolio wishes to improve the reproducibility of the work that we publish. This form provides structure for consistency and transparency in reporting. For further information on Nature Portfolio policies, see our Editorial Policies and the Editorial Policy Checklist.

Statistics

For all statistical analyses, confirm that the following items are present in the figure legend, table legend, main text, or Methods section.

n/a	Confirmed	
☐	☒	The exact sample size (*n*) for each experimental group/condition, given as a discrete number and unit of measurement
☐	☒	A statement on whether measurements were taken from distinct samples or whether the same sample was measured repeatedly
☐	☒	The statistical test(s) used AND whether they are one- or two-sided<br>*Only common tests should be described solely by name; describe more complex techniques in the Methods section.*
☐	☒	A description of all covariates tested
☐	☒	A description of any assumptions or corrections, such as tests of normality and adjustment for multiple comparisons
☐	☒	A full description of the statistical parameters including central tendency (e.g. means) or other basic estimates (e.g. regression coefficient) AND variation (e.g. standard deviation) or associated estimates of uncertainty (e.g. confidence intervals)
☐	☒	For null hypothesis testing, the test statistic (e.g. *F*, *t*, *r*) with confidence intervals, effect sizes, degrees of freedom and *P* value noted<br>*Give P values as exact values whenever suitable.*
☒	☐	For Bayesian analysis, information on the choice of priors and Markov chain Monte Carlo settings
☒	☐	For hierarchical and complex designs, identification of the appropriate level for tests and full reporting of outcomes
☒	☐	Estimates of effect sizes (e.g. Cohen's *d*, Pearson's *r*), indicating how they were calculated

Our web collection on statistics for biologists contains articles on many of the points above.

Software and code

Policy information about availability of computer code

Data collection	Data collection for each set of experiment is described in the corresponding section of the material and methods. Imaging data were collected using Metamorph® (Molecular Devices). Biochemistry data were collected using LI-COR Odyssey FC system (LI-COR) and ChemiDoc Touch system (Bio-Rad). Electrophysiology data were acquired using Clampex (Axon Instruments).
Data analysis	Imaging data were analyzed with Metamorph® (Molecular Devices). Dual-color super-resolution STORM data were analyzed with SAFe Neo software (Abbelight). Single molecule tracking data were analyzed with MATLAB (MathWorks). Electrophysiological data were analyzed using MiniAnalysis (Synaptosoft). All graphs, fits, and statistical tests were performed using GraphPad Prism.

For manuscripts utilizing custom algorithms or software that are central to the research but not yet described in published literature, software must be made available to editors and reviewers. We strongly encourage code deposition in a community repository (e.g. GitHub). See the Nature Portfolio guidelines for submitting code & software for further information.

Data

Policy information about availability of data

All manuscripts must include a data availability statement. This statement should provide the following information, where applicable:

- Accession codes, unique identifiers, or web links for publicly available datasets
- A description of any restrictions on data availability
- For clinical datasets or third party data, please ensure that the statement adheres to our policy

Data supporting the findings of this study are available within the main article, supplementary information files, and source data files. The mass spectrometry proteomics data have been deposited to the ProteomeXchange Consortium via the PRIDE partner repository with the dataset identifier PXD054623.

Research involving human participants, their data, or biological material

Policy information about studies with human participants or human data. See also policy information about sex, gender (identity/presentation), and sexual orientation and race, ethnicity and racism.

Reporting on sex and gender	*Use the terms sex (biological attribute) and gender (shaped by social and cultural circumstances) carefully in order to avoid confusing both terms. Indicate if findings apply to only one sex or gender; describe whether sex and gender were considered in study design; whether sex and/or gender was determined based on self-reporting or assigned and methods used. Provide in the source data disaggregated sex and gender data, where this information has been collected, and if consent has been obtained for sharing of individual-level data; provide overall numbers in this Reporting Summary. Please state if this information has not been collected. Report sex- and gender-based analyses where performed, justify reasons for lack of sex- and gender-based analysis.*
Reporting on race, ethnicity, or other socially relevant groupings	*Please specify the socially constructed or socially relevant categorization variable(s) used in your manuscript and explain why they were used. Please note that such variables should not be used as proxies for other socially constructed/relevant variables (for example, race or ethnicity should not be used as a proxy for socioeconomic status). Provide clear definitions of the relevant terms used, how they were provided (by the participants/respondents, the researchers, or third parties), and the method(s) used to classify people into the different categories (e.g. self-report, census or administrative data, social media data, etc.) Please provide details about how you controlled for confounding variables in your analyses.*
Population characteristics	*Describe the covariate-relevant population characteristics of the human research participants (e.g. age, genotypic information, past and current diagnosis and treatment categories). If you filled out the behavioural & social sciences study design questions and have nothing to add here, write "See above."*
Recruitment	*Describe how participants were recruited. Outline any potential self-selection bias or other biases that may be present and how these are likely to impact results.*
Ethics oversight	*Identify the organization(s) that approved the study protocol.*

Note that full information on the approval of the study protocol must also be provided in the manuscript.

Field-specific reporting

Please select the one below that is the best fit for your research. If you are not sure, read the appropriate sections before making your selection.

☒ Life sciences ☐ Behavioural & social sciences ☐ Ecological, evolutionary & environmental sciences

For a reference copy of the document with all sections, see nature.com/documents/nr-reporting-summary-flat.pdf

Life sciences study design

All studies must disclose on these points even when the disclosure is negative.

Sample size	Sample size was determined based on previous experiments and are described in each figure legend. Datapoints are shown and numbers as well as experimets are indicated in the ledgends. No precalculations were performed. For imaging experiments involving transfected neurons, all positive cells were acquired and analyzed, except for super-resolution and electrophysiology experiments were fewer cells were acquired and analyzed. Experiments were repeated at least 2-3 times. For experiments involving cell lines, at least 20 cells per condition and per experiments were acquired and analyzed. Presented samples sizes were considered sufficient based on the reproducibility of the results across multiple biological independent preparations.
Data exclusions	Electrophysiological recordings with Rs higher than 30 MΩ and changes > 20% were discarded. FRAP recovery curves displaying a negative slope were discarded. Outlier tests in GraphPad Prism was systematically performed when analysing datasets, and outliers were removed from distributions.
Replication	Replication was systematically performed with independent biological preparations. The number of replicates are indicated in each figure legend.

Randomization	Sample randomization was performed when replicating experiments to ensure that the sequence or timing of different acquisitions did not affect experiment outcome.
Blinding	Bliding was systematically performed for data analysis, but not systematically for data collection because acquisition parameters had to be defined to apply to the different conditions successfully.

Reporting for specific materials, systems and methods

We require information from authors about some types of materials, experimental systems and methods used in many studies. Here, indicate whether each material, system or method listed is relevant to your study. If you are not sure if a list item applies to your research, read the appropriate section before selecting a response.

Materials & experimental systems

n/a	Involved in the study
☐	☒ Antibodies
☐	☒ Eukaryotic cell lines
☒	☐ Palaeontology and archaeology
☐	☒ Animals and other organisms
☒	☐ Clinical data
☒	☐ Dual use research of concern
☒	☐ Plants

Methods

n/a	Involved in the study
☒	☐ ChIP-seq
☒	☐ Flow cytometry
☒	☐ MRI-based neuroimaging

Antibodies

Antibodies used	mouse-α-PSD-95 (ThermoFischer Scientific, clone 7E-1B8, 1:400), guinea-pig-α-VGluT-1 (AB5905, Merck Chemi-cals, 1:1000), mouse-α-GluA1/2 (Synaptic Systems, 182411, 1:100), mouse-α-gephyrin (SynSys, 147111, 1:1000), rabbit-α-RIM1/2 (SynSys, 140213, 1:500), mouse-a-synapsin-1 (Synaptic System, 106011, 1:500), sheep-α-LRRTM2 (R&D Systems, AF5589, 1:200), mouse-α-β-actin (Sigma-Aldrich #A5316), rabbit-a-streptavidin (Rock-land, 100-4195), rabbit-a-GFP (Abcam Ab-290), AlexaFluor®647-Goat-α-Human-IgG (Jackson ImmunoResearch Laboratories, 109-605-098), horseradish peroxidase (HRP)-donkey anti-secondary antibody (Jackson Immu-noresearch; 713-035-003), IRDye® 680RD Streptavidin (LI-COR #926-68079), IRdye-800CW goat-a-rabbit (LI-COR # 926-32211), goat-α-mouse-AlexaFluor488 (ThermoFischer Scientific, #A11001 1:800), goat-α-guinea-pig-DyLight405 (Abberior, #106-475-003, 1:800), goat-α-mouse-IgG2a-AlexaFluor647 (ThermoFisher Scientific #A21241), goat-a-rabbit-AlexaFluor647(ThermoFisher Scientific #A21244) and CF®597R-goat-α-rabbit (biotium #20797).
Validation	All antibodies used in this study were commercially developed and validated by the companies. All information on their application field, dilution, validation, etc. can be found on the corresponding websites with the references provided here.<br>mouse-α-PSD-95 (ThermoFischer Scientific, clone 7E-1B8, 1:400), guinea-pig-α-VGluT-1 (AB5905, Merck Chemi-cals, 1:1000), mouse-α-GluA1/2 (Synaptic Systems, 182411, 1:100), mouse-α-gephyrin (SynSys, 147111, 1:1000), rabbit-α-RIM1/2 (SynSys, 140213, 1:500), mouse-a-synapsin-1 (Synaptic System, 106011, 1:500), sheep-α-LRRTM2 (R&D Systems, AF5589, 1:200), mouse-α-β-actin (Sigma-Aldrich #A5316), rabbit-a-streptavidin (Rock-land, 100-4195), rabbit-a-GFP (Abcam Ab-290), AlexaFluor®647-Goat-α-Human-IgG (Jackson ImmunoResearch Laboratories, 109-605-098), horseradish peroxidase (HRP)-donkey anti-secondary antibody (Jackson Immu-noresearch; 713-035-003), IRDye® 680RD Streptavidin (LI-COR #926-68079), IRdye-800CW goat-a-rabbit (LI-COR # 926-32211), goat-α-mouse-AlexaFluor488 (ThermoFischer Scientific, #A11001 1:800), goat-α-guinea-pig-DyLight405 (Abberior, #106-475-003, 1:800), goat-α-mouse-IgG2a-AlexaFluor647 (ThermoFisher Scientific #A21241), goat-a-rabbit-AlexaFluor647(ThermoFisher Scientific #A21244) and CF®597R-goat-α-rabbit (biotium #20797).

Eukaryotic cell lines

Policy information about cell lines and Sex and Gender in Research

Cell line source(s)	COS-7 cells (ECACC, #87021302)
Authentication	Cell lines were authenticated by limited passage number and morphology.
Mycoplasma contamination	All cells tested negative for mycoplasma.
Commonly misidentified lines (See ICLAC register)	No commonly misidentified cell lines were used in the study.

Animals and other research organisms

Policy information about studies involving animals; ARRIVE guidelines recommended for reporting animal research, and Sex and Gender in Research

Laboratory animals	LRRTM2flox/flox mouse line was outsourced to Ingenious Targeting Laboratory. Floxed mice were maintained on C57BL/6J

Laboratory animals	background (Charles River). All experiments were performed (P0-P5) according to European guide for care and use of laboratory animals and animal care guidelines issued by the animal experimental committee of Bordeaux Universities (CE50; A5012009).
Wild animals	No wild animals were used in the study.
Reporting on sex	Sex and gender were not considered in the study
Field-collected samples	No filed-collected samples were used in the study.
Ethics oversight	All experiments were performed according to European guide for care and use of laboratory animals and animal care guidelines issued by the animal experimental committee of Bordeaux Universities (CE50; A5012009).

Note that full information on the approval of the study protocol must also be provided in the manuscript.

Plants

Seed stocks	*Report on the source of all seed stocks or other plant material used. If applicable, state the seed stock centre and catalogue number. If plant specimens were collected from the field, describe the collection location, date and sampling procedures.*
Novel plant genotypes	*Describe the methods by which all novel plant genotypes were produced. This includes those generated by transgenic approaches, gene editing, chemical/radiation-based mutagenesis and hybridization. For transgenic lines, describe the transformation method, the number of independent lines analyzed and the generation upon which experiments were performed. For gene-edited lines, describe the editor used, the endogenous sequence targeted for editing, the targeting guide RNA sequence (if applicable) and how the editor was applied.*
Authentication	*Describe any authentication procedures for each seed stock used or novel genotype generated. Describe any experiments used to assess the effect of a mutation and, where applicable, how potential secondary effects (e.g. second site T-DNA insertions, mosiacism, off-target gene editing) were examined.*
